# Precision in Dermatology: Developing an Optimal Feature Selection Framework for Skin Lesion Classification

**DOI:** 10.3390/diagnostics13172848

**Published:** 2023-09-02

**Authors:** Tallha Akram, Riaz Junejo, Anas Alsuhaibani, Muhammad Rafiullah, Adeel Akram, Nouf Abdullah Almujally

**Affiliations:** 1 Department of Electrical and Computer Engineering, COMSATS University Islamabad, Wah Cantt Campus, Islamabad 45040, Pakistan; 2 Department of Information Systems, College of Computer Engineering and Sciences, Prince Sattam bin Abdulaziz University, Al-Kharj 11942, Saudi Arabia; ah.alsuhaibani@psau.edu.sa; 3 Department of Mathematics, COMSATS University Islamabad, Lahore Campus, Lahore 54000, Pakistan; 4 Department of Information Systems, College of Computer and Information Sciences, Princess Nourah bint Abdulrahman University, Riyadh 11671, Saudi Arabia; naalmujally@pnu.edu.sa

**Keywords:** convolutional neural networks, feature selection, transfer learning, feature fusion, gray wolf optimization, deep learning, skin lesion

## Abstract

Melanoma is widely recognized as one of the most lethal forms of skin cancer, with its incidence showing an upward trend in recent years. Nonetheless, the timely detection of this malignancy substantially enhances the likelihood of patients’ long-term survival. Several computer-based methods have recently been proposed, in the pursuit of diagnosing skin lesions at their early stages. Despite achieving some level of success, there still remains a margin of error that the machine learning community considers to be an unresolved research challenge. The primary objective of this study was to maximize the input feature information by combining multiple deep models in the first phase, and then to avoid noisy and redundant information by downsampling the feature set, using a novel evolutionary feature selection technique, in the second phase. By maintaining the integrity of the original feature space, the proposed idea generated highly discriminant feature information. Recent deep models, including Darknet53, DenseNet201, InceptionV3, and InceptionResNetV2, were employed in our study, for the purpose of feature extraction. Additionally, transfer learning was leveraged, to enhance the performance of our approach. In the subsequent phase, the extracted feature information from the chosen pre-existing models was combined, with the aim of preserving maximum information, prior to undergoing the process of feature selection, using a novel entropy-controlled gray wolf optimization (ECGWO) algorithm. The integration of fusion and selection techniques was employed, initially to incorporate the feature vector with a high level of information and, subsequently, to eliminate redundant and irrelevant feature information. The effectiveness of our concept is supported by an assessment conducted on three benchmark dermoscopic datasets: PH2, ISIC-MSK, and ISIC-UDA. In order to validate the proposed methodology, a comprehensive evaluation was conducted, including a rigorous comparison to established techniques in the field.

## 1. Introduction

Cancer is caused by the uncontrolled multiplication of abnormal cells. Human cells frequently possess the capacity to replicate and divide, and abnormally replicated cells can spread through the lymphatic and vascular systems, wreaking havoc on a healthy body [1]. The five main forms of cancer recognized by Stanford Health Care (SHC) are carcinoma, sarcoma, lymphoma, leukemia, and myeloma. Most cases of the malignant melanoma variety belong to the class of carcinoma [2,3]. One of the most lethal and prevalent cancers in the world is skin cancer [4]. Sunlight has been linked to skin cancer in recent studies, because radiation is the main source of these rays; however, some artificial light also causes DNA damage to skin cells. Skin cancer can also be caused by genetic abnormalities or diseases that run in families [5].

**Figure 1:**
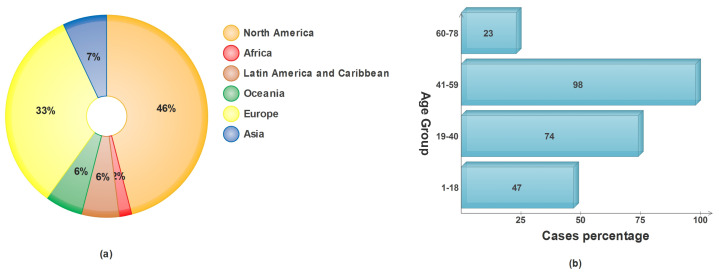
Epidemiological data on skin cancer: (**a**) WHO projections for skin cancer in 2022; (**b**) average accumulated sun exposure vs. age groups.

Skin cancer in any of its forms affects an estimated 9500 people every day in the United States alone, as reported by the American Cancer Society (ACS) [6]. In the year 2022, a total of 99,780 incidents of melanoma were identified, with 57,180 cases affecting males and 42,600 cases affecting females [7]. It is anticipated that almost 5080 men and 2570 women will lose their lives to this disease this year. Incidences of malignant melanoma have been steadily climbing over the course of the past few decades, with rates varying according to the ages of the people affected [8]. The percentage of skin cancer caused by sun exposure in different age categories and the number of cases diagnosed on each continent are presented in Figure 1.

With a cautious prediction of 17,756 new cases in 2022, the Australian Institute of Health and Welfare (AIHW) predicts that both melanoma and non-melanoma cases will rise to the third-most-often diagnosed cancer type in Australia. Diagnosis rates are as follows: 58.5% male and 41.5% female [9,10]. Clinical examinations often involve a practitioner or dermatologist observing a suspect’s skin in a series of phases. The most common method is the ABCDE rule, in which the appearance of the lesion (symmetry, border, color, and diameter) and evolution of the lesion are observed [11]. Assessment of the skin’s appearance is heavily influenced by the observer’s eyesight, which varies from person to person. Such observational screening for skin lesions has significant limitations and cannot provide an accurate diagnosis. Despite the best efforts of dermatologists, a recent study found that only 80% of cases were correctly diagnosed [12].

Machine learning methods have been widely implemented in several domains, including activity recognition [13], experimental systems [14], embedded systems [13], and public health care [2], for nearly two decades. The employment of these cutting-edge methods has simplified the entire process of disease detection and diagnosis [15]. Computer-aided diagnostic (CAD) systems [16] have the potential to replace conventional surgical assessment methods based on auto-generated feature analysis using machine learning approaches [17]. Therefore, scientists are certain that machine learning techniques will eventually replace conventional approaches to evaluating surgical procedures [18,19,20]. Computer vision techniques potentially assist medical practitioners in efficiently diagnosing skin cancer within a reduced time frame. This study primarily focused on the use of feature fusion and selection methods together. The research presented in this study makes two primary contributions: firstly, the introduction of a bio-inspired feature selection strategy aimed at addressing the challenges posed by the *"curse of dimensionality"* and over-fitting; secondly, to enhance the efficacy of the extracted features, a fusion mechanism is employed that leverages the complementary strengths of four pretrained models.

The subsequent sections of the article are structured as follows: Section 2 of the paper encompasses the literature review, which is subsequently followed by the problem statement and the contributions made in Section 3. Section 4 comprises two distinct subsections, the first of which elucidates the datasets and models employed, while the latter expounds upon the proposed framework. The final section of the paper elucidates the simulation and analysis, providing a comprehensive account of the obtained results. The final section of the article, Section 6, serves as a conclusion and provides an overview of potential future research directions.

## 2. Literature Review

**Figure 2:**
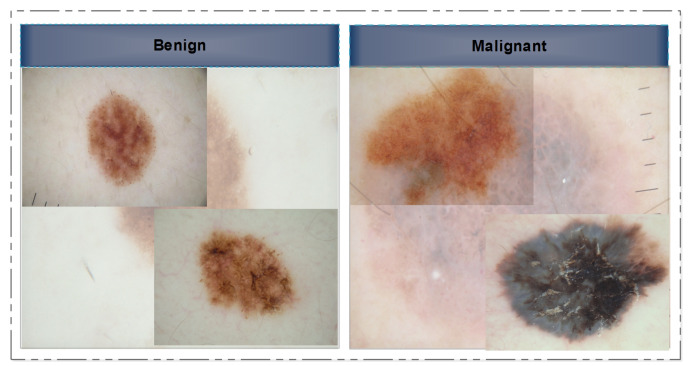
Selected skin lesion samples showing the benign class (**left**) and the malignant class (**right**).

In this section, we provide a concise literature assessment of work done on skin lesion classification using CNNs. In a few cases, the classification frameworks were applied directly to the provided image samples, while in others, the images were initially pre-processed before being subjected to the main processing steps. We begin with a discussion of non-traditional or deep-model approaches to image classification. Several researchers have approached this issue by considering it as a binary classification problem, where the images are categorized into two primary classes: malignant and benign. Several image samples are presented to the readers as references in Figure 2. However, a small number of researchers even employed seven classes.

The research of [21] employed deep learning models for the automatic categorization of multi-class skin lesions. Their presented algorithm was based on the deep convolutional neural network (DCNN), which contains several stacked layers and variable filter sizes. The authors claimed to have attained 90.1% precision, 93.23% sensitivity, and 91.1% specificity on the ISIC-17 dataset. The proposed algorithm demonstrated superior performance in comparison to various alternative methods—particularly in the context of low-resolution images. Similarly, the authors in [22] proposed a DCNN framework, to categorize skin lesions images into seven different classes that were subsequently consolidated into two overarching classes: healthy and cancerous. One limitation of this study was the occasional inability to directly consolidate classes into a smaller number. An evaluation of different CNN architectures was undertaken in the work by [23], in which the authors utilized different configurations of 12 CNN models, and set seven different classifiers. The DenseNet201 combined with the KNN classifier resulted in the best F1-score, accuracy, recall, and precision values.

The methodology proposed by Bi et al. [24] employed a hyper-connected convolutional neural network (HcCNN), to classify skin lesion images. The proposed approach involved the implementation of a deep hierarchical convolutional neural network (HcCNN) that incorporated a multi-scale attention block. This integration enabled the model to effectively capture and utilize the visual characteristics present in both dermoscopy and clinical skin cancer image datasets. The method proposed in this study demonstrated a slightly reduced level of performance, in terms of accuracy (82.70%), sensitivity (68.18%), specificity (84.62%), and precision (75.98%). Similarly, the work of [25] addressed the classification of high-resolution images and class variation present in real datasets. They proposed a framework known as patch-based attention architecture (pretrained CNNs). The outlined algorithm provided a global context in between low- and high-resolution regions. The mean values of the achieved sensitivity, specificity, and F1-score were 73.3%, 96.3%, and 85.3%, which were quite low, as some of the methods achieved better results compared to the existing techniques. In [26], the authors outlined a method of accelerating the performance of classifying the skin lesions, by using generated adversarial networks (GANs) based on data augmentation technology. On the dataset ISIC-2018, the obtained accuracy, specificity, sensitivity, and average precision were, respectively, 95.25%, 96.61%, 83.21%, and 83.11%. Despite the authors’ claims that their acquired parameters were better than the CNN model, improvements are still needed to their multi-class accuracy, specificity, and sensitivity. The presented algorithm was effective only for skin lesion regions with high resolution and better diversity.

The proposed framework of [27] combined a skin lesion boundary segmentation (conducted using a full-resolution convolutional network) stage and a multiple skin cancer lesions classification stage. Then, a CNN, such as ResNet-50, Inception-v3, DenseNet-201, and Inception-ResNet-v2, was employed. The maximum achieved values of specificity, sensitivity, accuracy, true-negative rate (F1-score), and area under the curve were 80.62%, 75.67%, 75.75%, and 81.57%, respectively, on the ISIC 2017 dataset. In their study, Behara et al. [28] presented a model for categorizing skin lesions, which was founded on the utilization of deep convolutional generative adversarial networks (DCGAN). The methodology employed in this study yielded real-time images that were suitable for training purposes. Furthermore, these generated images were further improved by the application of different image processing techniques. The classification task was executed by the final layer of the discriminator, which predicted the desired class. The claimed performance metrics on the ISIC2017 dataset included accuracy of 99.83%, and precision and recall rates of 99%. While the generated images exhibited a certain degree of resemblance to genuine images, they were found to be deficient in terms of richness and diversity. The authors additionally provided a restricted level of control over the hyperparameters. The proposed method in [29] classified the cancer lesion by using ensembles of CNN models known as multi-resolution EfficientNets with metadata. Lesion classification was conducted using EfficientNets, SENet, and ResNet WSI. The achieved values of the area under the curve were in the range 77.5–96% and those of sensitivity were in the range 0.283–71%, obtained on the ISIC-2019 dataset. In [30], the authors proposed a cascade knowledge diffusion network (CKDNet) that transferred and accumulated the information gathered from various sub-tasks, to increase the efficiency of segmenting and classifying cancer images. They reported better performance without ensemble approaches or external datasets and every time neural networks needed to be trained, which took a lot of time: hence, in some applications, this could be a drawback. On the contrary, the authors in [31] proposed an approach for multi-label ensemble multi-class classification of skin cancer images. The efficiency of this method was only compared to that of the specialist’s advice.

The integration of conventional and contemporary frameworks is a subject of interest among researchers in the field. The work of [32] used a wavelet-based CNN model. The method decomposed the input image into seven different directional sub-bands. The sub-band images were fed to eight pretrained CNNs, as an input, to generate eight probabilistic classifiers. The efficiency of the proposed method was evaluated for seborrheic keratosis and melanoma classification. The authors concluded that the model I-GR0235 outperformed other models, in terms of performance. But the achieved values of accuracy (83%), the receiver operating characteristic curve (91%), and sensitivity (13%) were not convincing. Similarly, the authors in [33] presented a multi-level, multi-class algorithm implemented by available machine learning tools and advanced deep learning methods based on the divide and conquer rule. They achieved specificity, sensitivity, precision, and accuracy of 98.45%, 87.21%, 98.25%, and 92.82%, respectively, for the testing phase.

Researchers are still developing conventional methods for classifying cutaneous lesions. The work proposed in [34] was based on sparse representation for classification of lesion images. The developed algorithm produced discriminating sparse codes representing the features in a high-dimensional feature set. The reported values of sensitivity, accuracy, and specificity were 96.61%, 94.83%, and 93.31%, respectively, on the ISIC 2016 dataset. Similarly, the approach in [35] utilized a network called the self-supervised topology clustering network (STCN), to transform an invariant network, using a self-supervised modularity clustering algorithm based on the principles of topology analysis. The efficacy of the proposed STCN was compromised, due to its inability to effectively filter negative sample images, resulting in a decrease in classification performance. Additionally, the hand-crafted features included in the STCN also suffered from this limitation. There are some other applications in the medical imaging domain that have adopted hybrid techniques [36,37,38,39,40].

The literature review is concisely presented in Table 1. The given indices are PRC (precision), SEN (sensitivity), ACC (accuracy), SPC (specificity), AUC (area under the curve), F1-S (F1-score), Ppv (+ve predictive value), and Hm (Harmonic Mean).

## 3. Problem Statement and Contributions

Over the past few years, computer-aided detection (CAD) systems have become increasingly important in the detection and assessment of skin lesions. Nevertheless, the classification process is hindered by various limitations at both the image level—including low-contrast lesion regions, skin flakes, the presence of hair, and air bubbles—and at the feature level, such as redundant or missing feature information. Consequently, achieving accurate classification becomes challenging. The presence of these undesirable characteristics has a direct or indirect impact on the segmentation and classification processes, leading to a decline in the overall performance of the system. Hence, it is imperative to tackle these issues at various stages, to establish a resilient framework for detection and classification. This study primarily examined the impact of feature-level information on the ultimate classification outcome. Following the feature extraction phase, conventional feature selection techniques frequently encounter challenges related to increased computational cost and diminished accuracy. Hence, in order to address the aforementioned issue, hybrid metaheuristic algorithms were introduced, to enhance performance. Two main contributions can be drawn from the findings of this study:

- Introduction of a bio-inspired feature selection strategy called the entropy-controlled gray wolf optimization algorithm, which is designed to resolve the challenges posed by the “curse of dimensionality” and over-fitting. This technique emphasizes identifying the most discriminant features, to mitigate these issues.
- Adoption of a fusion method, to combine the strengths of four pretrained models, so as to improve the efficiency of the extracted features.

Given a database of dermoscopic images, we had to attribute a label to each and every image, classifying them as either benign or malignant. We let an image $I\subset R^{\left(i\times j\times k\right)}$ be a dermoscopic image for a given database $D^{\kappa }$. The set of images were $\left\{\left(I_{1}^{\kappa }\right),\left(I_{2}^{\kappa }\right),\cdots ,\left(I_{L}^{\kappa }\right)\right\}\subset \left\{D^{K}\right\}\in R^{\left(1\times K\right)}$. For a given image, the number of channels $L\subset I_{l}^{p}$ were fixed to be three, and the number of classes *C* were provided by the user. Therefore, for each image, the extracted features, $\phi \in R^{\left(r\times c\right)}$, were later subjected to the classifier for the label assignment, $\tilde{\phi }$, against each image. The cascaded system, which consisted of a series of steps, including feature fusion and selection, was ultimately represented as

(1) $$\tilde{\phi }≜\left(\phi_{m}^{f},\phi^{fs},\tilde{\kappa }\left(\phi^{fs}\right)\right)\in R^{\left(r\times c\right)},$$

where $\phi^{f}$ denoted the features extracted after employing the transfer learning, $\phi^{fs}$ depicted the fused feature set from fully connected layers of different architectures, and $\tilde{\kappa }\left(\phi^{fs}\right)$ was the representation of the selected feature set as the output of a hierarchical structural design.

## 4. Material and Methods

### 4.1. Convolutional Neural Networks (CNNs)

**Figure 3:**
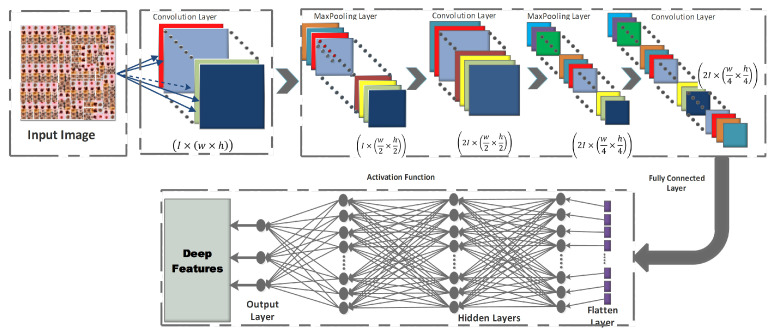
Basic Architecture of a CNN.

CNNs are the most spectacular versions of deep feedforward neural networks used for feature detecting and classifying [16,45]. Each neuron in a CNN is linked to a group of other neurons in the higher layer, using a feedforward technique. Convolution, pooling, and fully linked layers make up the three main sub-blocks of a CNN’s fundamental architecture, as depicted in Figure 3.

- *Convolution layer*: In the CNN architecture, this is the most basic and crucial element. The primary goal of it is to identify and extract local feature sets from an input image, $I^{\kappa }\subset D^{\kappa }$. Let the image database be divided into training ($D_{tr}^{K1}$) and testing databases $D_{ts}^{K2}$, where {$D^{K1},D^{K2}\}\;\subset \;D^{K}$. The training samples are represented as $Y=\{y_{1},y_{2},\cdots ,y_{n}\}$, where *n* denotes the training image database size. For each given input image, the resulting output image is $Z=\{z_{1},z_{2},\cdots ,z_{n}\}$, where $z_{p}\in \left\{1,2,\cdots ,C\right\},C$ signifies the class number. The convolutional layer consists of a kernel filter that goes through each pixel of the input image as $I^{\left(i\times j\times k\right)}*H^{\left(i^{\prime }\times j^{\prime }\times k\right)}$. The local feature set $F\in F_{l}$ is obtained, based on the following equation:
  (2) $$F_{i}^{l}=\sigma \left(\sum_{i=1}^{n}x_{i}^{l-1}\times \delta_{i}^{l}+b_{l}^{j}\right),$$
  where $F_{i}^{l}$ denotes the output feature map for that particular layer, where $l;\delta_{i}^{l}+b_{l}^{j}$ are the trainable parameters for the layer, and where l;σ(.) is the activation function.

#### 4.1.1. Pretrained CNN Models

In this study, we utilized four State-of-the-Art pretrained models for feature extraction, including DarkNet53, InceptionV3, InceptionResNetV2, and DenseNet201. There are various proposed sets of CNN architectures for computer vision applications. This decision was made based on their performance, number of parameters, and Top-1 accuracy.

- *Inception-V3*: The two essential components of Inception-V3 are feature extraction and classification. It is trained using the ImageNet database. Using inception units, an Inception-framework V3 can increase a network’s depth and width while also reducing its computing load.
- *Inception-ResNet-V2*: As with the development of Inception-V3, Inception-ResNet-V2 is likewise trained using the ImageNet database. It combines the ResNet module and inception. The other connections enable bypass in the model, which strengthens the network. The computational prowess of the inception units and the optimization leverage provided by the residual connections are combined in Inception-ResNet-V2.
- *DenseNet-201*: The ImageNet database is also used to train DenseNet-201. It is built on an advanced connectivity scheme that continuously integrates all of the output properties in a feedforward manner. Furthermore, it strengthens feature propagation, decreases the number of input and functional parameters, and mitigates the problem of vanishing gradient.

#### 4.1.2. Datasets

In this study, we carried out our simulations on the three publicly available benchmark datasets:

- $PH^{2}:$ consists of 200 RGB images, divided into 160 benign and 40 melanoma image samples. The database is maintained by the Hospital Pedro Hispano, Matsinhos, through clinical observation using a dermoscope. The real physician’s response is also provided, i.e., normal, melanoma, or typical nevus.
- *ISIC-MSK:* the other database incorporated here is the International Skin Imaging Collaboration (ISIC). It includes 225 RGB dermoscopic image samples obtained from different well-reputed international cancer institutes, captured by various modalities.
- *ISIC-UDA:* is another publicly accessible dataset for the characterization and study of skin cancer (total images: 2750; training images: 2200; testing samples: 550). It contains three cancer types: melanoma, keratosis, and benign; but, since keratosis is a fairly common benign skin indication, the images can be divided into two classes: malignant and benign.

For evaluation purposes, dermatologists manually labeled all the datasets. Table 2 displays the distribution of images within the previously mentioned datasets.

### 4.2. Proposed Framework

**Figure 4:**
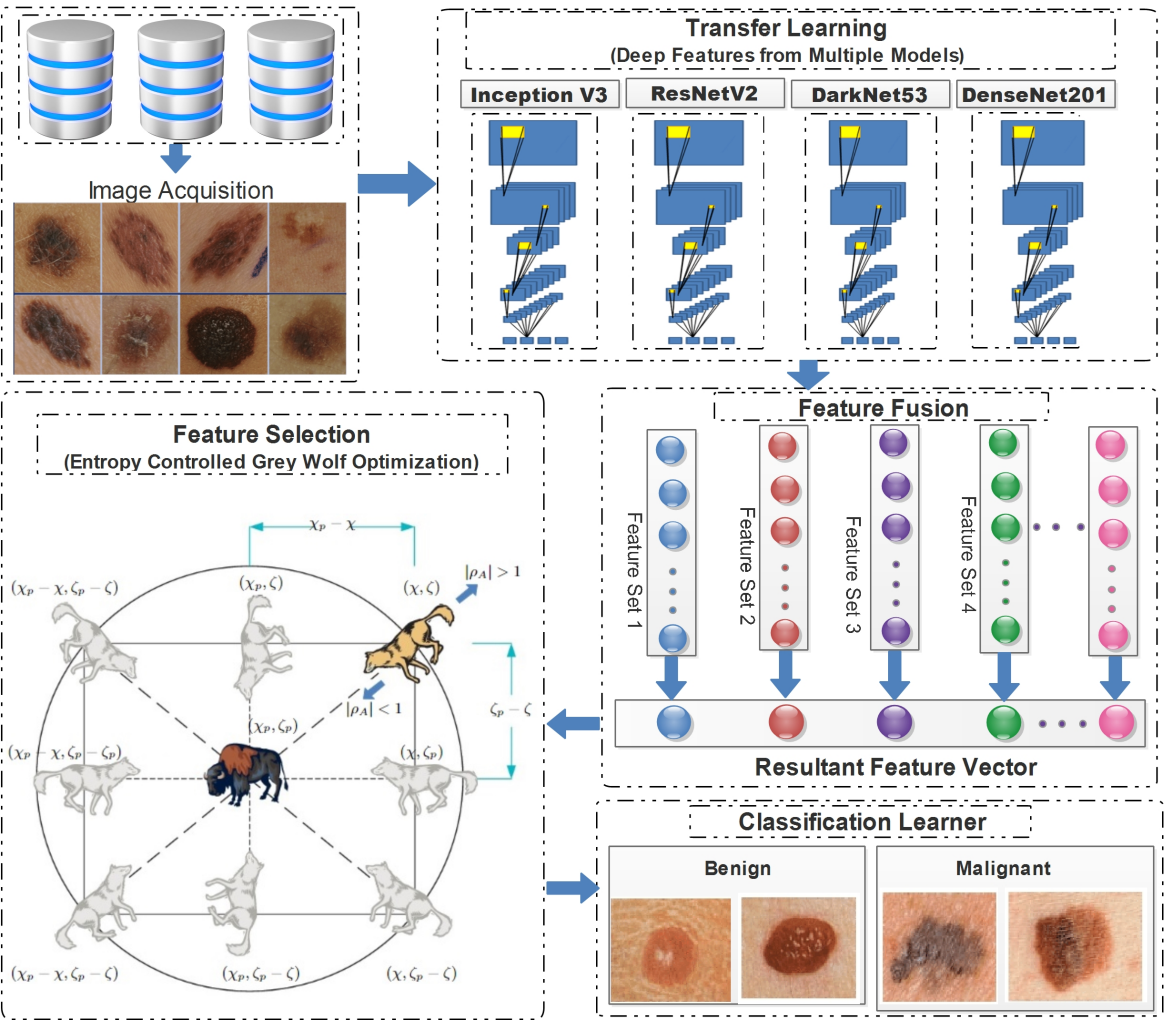
Detailed illustration of proposed skin lesion classification framework.

In this study, a conventional hierarchical approach was employed, encompassing feature extraction, and concluding with the final classification. The proposed framework employed transfer learning, to extract deep features from pretrained models. Subsequently, the extracted features were combined in a predetermined order, and these combinations were then subjected to the proposed feature selection method. The feature vectors obtained at the end of the process were subsequently employed for classification purposes. Figure 4 demonstrates the detailed flow of the proposed framework, from the image acquisition to the final classification.

#### 4.2.1. Transfer Learning

Convolutional algorithms operate under the assumption that the feature sets of both the training and testing datasets are nearly identical, allowing for straightforward estimation. Although numerous pretrained models have undergone extensive training on general image datasets, they may not be optimal for specialized applications. Transfer learning (TL) is a viable approach, as it effectively classifies images using a limited number of training instances, even in scenarios where acquiring real-world data poses challenges. The optimal performance of transfer learning is achieved when the input and output source databases exhibit a significant degree of dispersion, in terms of their sizes, thereby ensuring a diverse source domain.

Consider a source domain, $\Psi_{s}=\left\{\left(x_{1}^{s},y_{1}^{s}\right),\left(x_{2}^{s},y_{2}^{s}\right),\cdots \left(x_{n}^{s},y_{n}^{s}\right)\right\}$, where

$\left(x_{i}^{s},y_{i}^{s}\right)\in R^{2}$; with particular learning assignments, $L_{S}$, and target domain $D_{T}=\left\{\left(x_{1}^{T},y_{1}^{T}\right),\left(x_{2}^{T},y_{2}^{T}\right),\cdots \left(x_{n}^{T},y_{n}^{T}\right)\right\}$ having the learning assignment as $L_{S},\left(x_{i}^{T},y_{i}^{T}\right)\in R^{2}$, where 1≤i≤n.

Let us consider, for a given dataset, the number of image samples are $D_{Tl}^{T}$ and the model is trained over a large dataset $D_{s}^{LS}$, where $D_{Tl}^{T}$≪$D_{s}^{LS}$, and their labels are $y_{Tl}^{T}$ and $y_{s}^{LS}$. The primary objective of transfer learning (TL) is to enhance the learning effectiveness of the target function $\tilde{\phi }$, by leveraging the information derived from both the source dataset $D_{s}^{LS}$ and the target dataset $D_{T}^{Tl}$.

#### 4.2.2. Feature Fusion

The availability of highly discriminant information is a crucial factor in enhancing classification accuracy. The presence of redundancy and irrelevant information not only diminishes the accuracy of classification but also imposes a greater computational load. Furthermore, the likelihood of attaining a high level of classification accuracy through the sole utilization of a standard feature extraction approach is quite low. Hence, a methodology for feature fusion has been selected, which not only generates a comprehensive information vector but also leads to an increase in redundancy [46]. In order to address this issue, the utilization of feature fusion in conjunction with feature selection algorithms is employed. In this study, we integrated the extracted set of features obtained from the chosen pretrained models, following the implementation of transfer learning. It was supposed that for a given set of features extracted from the selected model after applying transfer learning, $\phi_{m}^{f}=\left\{\phi_{1}^{f},\;\phi_{2}^{f},\;\phi_{3}^{f},\;\phi_{4}^{f}\right\}\in R^{\left(r\times n\right)}$. The dimensions for the extracted features were given as $\phi_{m}^{f}=\left\{\left(s\times 2\right),\left(s\times 1536\right),\left(s\times 1026\right),\left(s\times 1920\right)\right\}$, extracted from the fully connected and average pooling layers of all the selected models. The fusion process involved a sequential concatenation of feature vectors, where each new vector was embedded into the existing one. The resultant feature vectors were generated from the combination of all the extracted feature vectors. We let $FV_{1}=\phi_{1}^{f}$, $FV_{2}=\phi_{2}^{f}$, $FV_{3}=\phi_{3}^{f}$, and $FV_{4}=\phi_{4}^{f}$. The concatenated form followed the property given: $\phi_{m}^{f}:=\phi_{1}^{f}\;⊕\phi_{2}^{f}=R^{p}⊕R^{q}\to R^{p+q}\Rightarrow \phi_{m}^{f}:=\left(\phi_{1}^{f},\phi_{2}^{f}\right)\to \left(u_{1},\cdots ,u_{p},v_{1},\cdots ,v_{q}\right)$, where $u_{k}\in \phi_{1}^{f}\subset R^{p}$ and $v_{l}\in \phi_{2}^{f}\subset R^{q}$. For the rest of the combinations, the property still held: $\phi_{m,1}^{fs}=\left[\phi_{2}^{f},\phi_{3}^{f}\right]$, $\phi_{m,2}^{fs}=\left[\phi_{3}^{f},\phi_{4}^{f}\right]$, $\phi_{m,3}^{fs}=\left[\phi_{2}^{f},\phi_{4}^{f}\right]$, $\phi_{m,4}^{fs}=\left[\phi_{2}^{f},\phi_{3}^{f},\phi_{4}^{f}\right]$, and $\phi_{m,5}^{fs}=\left[\phi_{1}^{f},\phi_{2}^{f},\phi_{3}^{f},\phi_{4}^{f}\right]$.

#### 4.2.3. Entropy-Controlled Gray Wolf Optimization

We employed entropy-controlled gray wolf optimization (GWO) [47], to achieve the desired result. In this section, we offer a brief but concise background on the method.

GWO is a metaheuristic optimization technique that imitates the hunting strategy and social organizational behavior of gray wolves. Like other metaheuristic algorithms, GWO possesses a distinct array of merits and demerits when compared to alternative optimization techniques. GWO exhibits several potential advantages in comparison to alternative evolutionary strategies, encompassing simplicity, efficient exploration and exploitation capabilities, reduced parameter requirements, and enhanced convergence speed. It may not exhibit superior performance compared to other optimization strategies, in all scenarios, but the outcomes achieved by this approach for the given application are remarkable. This framework counts on three primary steps: skirting the prey, encircling the prey, and finally attacking and hunting the prey. In GWO, the population is categorized into alpha (α) wolf, which is the leader of the gang, beta (β) wolf, the second leader, and delta (δ) wolf, which is the third leader. The beta wolf assists the alpha leader in making the decisions, and the delta wolf dominates the pack of wolves (ω). The hunting process is originally guided by three leaders, whereas the ω wolves only follow the leaders. The first step, i.e., the hunting step of the pack, is given as:

(3) $$\chi \left(t+1\right)=\chi_{p}\left(t\right)-\varrho_{A}\cdot \psi_{D},$$

where χ is the new position of the wolf, $\chi_{p}$ is current position, and $\varrho_{A}$ represents the coefficient vector. The variable $\psi_{D}$ depends on the current location of the prey $\left(\chi_{P}\right)$ and is defined as

(4) $$\psi_{D}=\left|\varrho_{c}\cdot \chi_{p}\left(t\right)-\chi \left(t\right)\right|.$$

here, $\varrho_{c}=2\cdot r$ is a random vector in the range [0,1]. Other coefficients can be further explored in the cited article [48]. If we assume that α, β, and δ are the three optimum solutions, the new position of the other wolves is modified using the following set of rules:

(5) $$\chi \left(t+1\right)=\frac{\chi_{1}+\chi_{2}+\chi_{3}}{3}.$$

here, $\varrho_{co}$ is the leader count—selected to be three. The position vectors are calculated by Equation (6):

(6) $$\begin{array}{c} \chi_{1}\;=\;|\chi_{\alpha }-|\varrho_{A}^{1}\cdot \psi_{D}^{\alpha }\left|\right|\\ \chi_{2}\;=\;|\chi_{\beta }-|\varrho_{A}^{2}\cdot \psi_{D}^{\beta }\left|\right|\\ \chi_{3}\;=\;|\chi_{\delta }-|\varrho_{A}^{3}\cdot \psi_{D}^{\delta }\left|\right|. \end{array}$$

The parameters $\chi_{\alpha }$, $\chi_{\beta }$, and $\chi_{\gamma }$ are the positions of α, β, and δ at *t* iteration. Other sets of parameters, including $\varrho_{A}^{1},\varrho_{A}^{2}$ and $\varrho_{A}^{3}$, are calculated using the reference article [48], such as

(7) $$\begin{array}{c} \psi_{D}^{\alpha }=\left|\varrho_{c}^{1}\cdot \chi_{\alpha }-\chi \right|\\ \psi_{D}^{\beta }=\left|\varrho_{c}^{2}\cdot \chi_{\alpha }-\chi \right|\\ \psi_{D}^{\delta }=\left|\varrho_{c}^{3}\cdot \chi_{\alpha }-\chi \right|, \end{array}$$

where $\varrho_{c}^{1}$, $\varrho_{c}^{2}$, and $\varrho_{c}^{3}$ are calculated as in [48]. GWO, in general, is utilized, to solve the continuous optimization problem. It optimizes by considering a set of random solutions; for each solution there is a vector that keeps the parameters’ values of the problem. The first step is to estimate the objective function value for each solution. For the current solution, the entropy-based fitness value is calculated on the basis of the total amount of information in an entire probability distribution. The population vector subjected to the entropy calculation offers a maximum information range. The fitness is therefore calculated using Shannon entropy:

(8) $$fit^{i}=-\sum_{p=1}^{n}\;\eta_{p}\;log_{2}\;\eta_{p},$$

where $\eta_{p}$ is the selected vector. Hence, each solution has one variable, to keep its objective value. There are vectors and parameters other than the aforementioned. These vectors and parameters store the objective function and location values of α, β, and δ wolves. These values are updated before updating the position of the wolves. The GWO algorithm keeps updating the solutions, using Equations (5)–(7).

**Figure 5:**
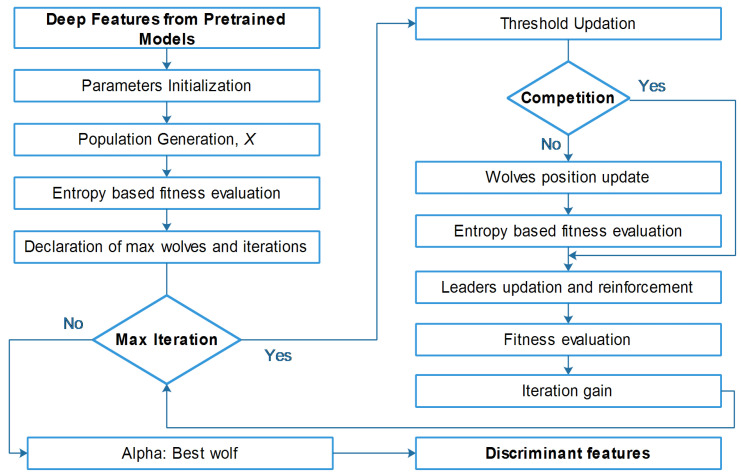
Detailed flow diagram of proposed entropy-controlled gray wolf optimization.

As mentioned above, we utilized GWO to solve the continuous optimization problem, but in the case of feature selection, we extended the work of [48] and embedded the concept of the entropy fitness function. A detailed flow of the proposed entropy-controlled gray wolf optimization algorithm is given in Figure 5.

## 5. Results and Analysis

The simulations awere carried out on three publicly available datasets, as shown in Table 2. Three families of contemporary classifiers—including support vector machines (SVM), k-nearest neighbors (KNN), and ensemble (ES)—were used for classification. The proposed framework was evaluated utilizing two configurations. In the initial configuration, the classification results were obtained without feature selection. In the second simulation setup, the proposed feature selection step was incorporated, to obtain the classification results. In order to make a fair comparison, we also evaluated the proposed framework alongside other classifiers. The training/testing ratio of 70:30 was selected, and hold-out cross-validation was chosen as the cross-validation technique. Table 3 provides all the necessary base parameters for the chosen classifiers. The selected parameters were selected based on the default values for all the Matlab sessions. In this study, we endeavored to employ a diverse range of classifiers, encompassing SVM, KNN, and ensemble methods. This selection was predicated on the previous empirical evidence of consistently achieving superior outcomes in comparison to alternative sets of classifiers for this specific application.

In the results section, we will discuss the impact of the feature vectors produced by applying transfer learning to four pretrained models. The flow was designed to take into account the feature combination vectors, their initial sizes, and the reduction percentage obtained after implementing the proposed feature selection algorithm.

**Figure 6:**
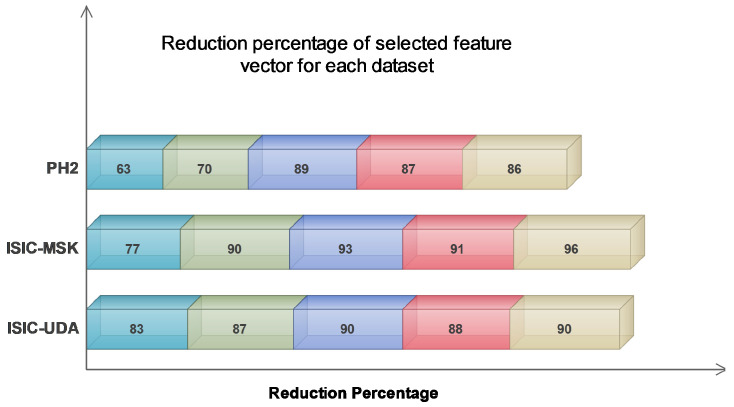
Comparison of reduction percentage for each selected dataset.

The findings presented in Table 4 demonstrate that the greatest reduction percentage was observed when all the extracted feature vectors (FV1–FV2–FV3–FV4) were combined. This suggests a high likelihood of redundant information. Despite the extent of the reduction achieved, the classification accuracy remained satisfactory. The average reduction percentage for the last feature combination, which included all feature vectors, was at its maximum value of 91.33%. By contrast, the average reduction percentages for the remaining feature combinations were 74.33%, 82.33%, 90.66%, and 88.66%, respectively. Additionally, an alternative manifestation of the impact can be observed in Figure 6, which illustrates that the greatest level of reduction was attained on the ISIC-MSK dataset. Based on the obtained results, we strongly believe that our proposed algorithm exhibits superior performance in handling large feature vectors, primarily due to its notable capability of effectively detecting and eliminating redundant information.

Based on the findings presented in Table 4, it can be inferred that the final combination exhibited the highest reduction rate. Building upon this observation, we proceeded to generate the testing accuracies, as well as other relevant parameters, such as sensitivity, specificity, false negative rate (FNR), false positive rate (FPR), and F1-score, in Table 5. We took accuracy as the primary measure, and we compared the performance specifically on this measure in addition to other measures. The FNR and the FPR of all the classifiers with greater accuracy and sensitivity were at their lowest, which clearly indicates the superior performance of these classifiers, including Fine KNN, Q-SVM, and ES-KNN. We focused primarily on accuracy in our comparisons, and we used this and other metrics to evaluate performance. Classifiers like Fine KNN, Q-SVM, and ES-KNN that had a high level of accuracy and sensitivity also had low levels of FNR and FPR, respectively, demonstrating their superior performance.

To provide a better insight, a fair comparison of the feature fusion approach with and without applying feature selection is also provided in Table 6. Three classifiers were employed, due to their superior accuracy and computational efficiency. The results demonstrate a noticeable enhancement in performance, following the implementation of the feature selection technique. When comparing the classification accuracy obtained using Fine KNN with and without feature selection, it is important to evaluate the impact of feature selection on the accuracy of the classification model. In the case of PH2, the maximum achieved accuracy was 98.89%, while the accuracy without feature selection was 85.22%. A discernible disparity of approximately 13% could be observed. Similar patterns were observed in other datasets. When considering the ISIC-MSK dataset, the accuracy rate was observed to be 99.01%. However, when utilizing Fine KNN with the same dataset, the accuracy rate decreased to 81.23%, although the classification accuracy achieved with other classifiers was 83.73%. Regarding ISIC-UDA, ES-KNN demonstrated an accuracy of 99.09%. Conversely, the maximum accuracy attained for the original fused feature vector was 89.74%. Additionally, in order to ensure a comprehensive evaluation, the classification accuracy of several established algorithms is also presented in Table 7. It is evident that our proposed method surpasses these existing algorithms, by exhibiting enhanced classification accuracy. Based on the statistical data, we hold the firm belief that our proposed feature selection techniques have the potential to yield exceptional results in various other applications.

## 6. Conclusions

Melanoma is widely acknowledged to be a highly fatal variant of skin cancer, with its occurrence demonstrating an increasing pattern in recent times [54]. Also in recent times, a number of computer-based methodologies have been put forth, with the aim of early detection and diagnosis of skin lesions. Despite having attained a certain degree of accomplishment, there persists a margin of error that is regarded as an unresolved research challenge within the machine learning community. The present study introduces an innovative framework for the categorization of skin lesions. The framework integrates deep features, in order to produce a feature vector that is highly discriminative, while simultaneously preserving the integrity of the original feature space. Our study utilized a selection of contemporary deep models—namely, Darknet53, DenseNet201, InceptionV3, and InceptionResNetV2—to perform feature extraction. Furthermore, the utilization of transfer learning was employed, to augment the efficacy of our methodology and, subsequently, feature selection was employed, to identify the most discriminant information. The approach demonstrated satisfactory performance in the majority of cases. However, it is important to note that the feature selection method may not be effective for feature vectors exhibiting maximum correlation. Furthermore, the inclusion of a pre-processing step has the potential to enhance accuracy even further.

There is room for improvement in a number of areas that could be investigated in further studies. Contrast enhancement, vision transformers (ViT), and feature selection are a few examples. Improved segmentation and classification accuracy could be achieved with the use of contrast enhancement techniques, by providing more refined pictures to the CNN/ViT models. Additionally, a dedicated CNN/ViT model may improve the system’s accuracy. As the feature selection mechanism is crucial in discarding superfluous data, other evolutionary methods and hybrid evolutionary methods could be proposed.

## Figures and Tables

**Table 1:** A comparative analysis of performance, techniques, and datasets in the literature, for various techniques and their evaluations.

Ref.	Year	Performance Parameters	Dataset	Remarks
[21]	2021	PRC = 94.0% AUC = 96.4%	ISIC-17,19	The proposed model had multiple layers and filter sizes, but fewer numbers of filters and parameters to classify the skin lesion images.
[41]	2018	SEN = 89.9% SPC = 92.1% F1-S = 90% Ppv = 91.3%	ISIC-17	An automatic approach to classifying melanoma, with the advantage of transforming the structural co-occurrence matrix (SCM) in an adaptive feature extractor, which helped the classification process to depend only on the input image as a parameter.
[25]	2019	SEN = 73.3% SPC = 96.3% F1-S = 85%	HAM	This research had two contributions: first, the efficient application of a high-resolution image dataset with pretrained state of the art architecture for classification; second, the high variation faced in the real image database.
[26]	2020	SEN = 83.2% ACC = 95.2% SPC = 96.6%	ISIC-18	A GAN-based data segmentation approach. The original generator’s style, control, and input noise structures were altered by the model. The classifier was generated by a pretrained DCNN, using the transfer learning method.
[23]	2020	PRC = 92.6% ACC = 92.5% F1-S = 92%	PH2	This work presented skin cancer lesion classification, using transfer learning and CNNs (as resource extractors). The method combined 12 CNN models with several different classifiers on PH2 and the ISBI-ISIC dataset.
[22]	2020	SEN = 73.7% ACC = 92.5% AUC = 91.2% Ppv = 74.1%	ISIC-17	A framework divided dermoscopic images in seven classes into two possible classes: positive/negative. The DCNN was trained, regarding this binary problem. The parameters regarding classification were later used to adjust for the multi-class categorization.
[30]	2021	SEN = 70.0% ACC = 88.1% SPC = 92.5% AUC = 90.5% Ppv = 73.8%	ISIC-17	The proposed framework was a series of coarse-level segmentation, categorization, and fine-level segmentation networks. Two feature-mixing modules were outlined, to accommodate the diffused feature set from starting segmentation, and to integrate the related knowledge learned, to help with fine-level segmentation.
[32]	2019	SEN = 17.0% ACC = 79.0% SPC = 95.0% AUC = 70.0%	ISIC-17	Seven separate directional sub-bands were created from gabor-wavelet-based DCNN, from input images. Subsequently, the output sub-band and input images were passed to eight parallel CNNs. To categorize the skin cancer lesion, the addition rule was used.
[34]	2019	SEN = 96.6% ACC = 94.8% SPC = 93.3%	ISIC-16	A kernel-sparse-representation-based method was proposed. A linear classifier and kernel-based metadata were both jointly adopted by the discriminative kernel sparse coding technique.
[33]	2020	SEN = 87.2% ACC = 92.8% ACC = 87.2%	ISIC	A multi-class, multi-level classification method focused on “divide and conquer” was presented. The algorithm was tuned using traditional NN tools and advanced deep learning methodologies.
[24]	2020	PRC = 75.9% SEN = 68.2% SPC = 84.6%	7 Point Checklist	This method used a hyper-connected CNN, by adding the visual properties of dermoscopy and clinical skin cancer images, and introducing a deep HcCNN with a multi-scale attention block.
[27]	2020	SEN = 75.7% ACC = 81.6% SPC = 80.6%	ISIC-17	The framework integrated a skin lesion boundary segment and a multiple skin lesions classification stage. Then, a CNN, such as Inception-v3, was employed.
[29]	2020	SEN = 71.0% AUC = 96.0%	ISIC-19	This method classified skin lesions with the help of the statistics of multi-resolution EfficientNets with metadata, using EfficientNets, SENet, and ResNet WSI.
[42]	2020	PRC = 91.3% ACC = 96.3% AUC = 98.1%	ISIC-18	The authors investigated the image size effect in classifying skin lesion images using pretrained CNNs. The performance of EfficientNetB0 &B1, and SetReNetXt50 was examined.
[35]	2021	ACC = 80.0%	ISIC-18	This research used a self-supervised topology clustering network (STCN), by transformation of an invariant model network by a modularity clustering algorithm.
[43]	2019	SEN = 91.7% ACC = 95.2% SPC = 97.9%	ISIC 2016	A recursive-feature-rejection-based layered structured multi-class image categorization was used. Before the classification, features such as shape and size, border non-uniformity, color, and texture of the skin lesion region were extracted.
[44]	2020	AUC = 92.1%	ISIC-17	The authors proposed a lesion classification method centered on mid-level features. Firstly, images were segmented, to identify the regions of interest; then, the pretrained DenseNet and ResNet were employed, to extract the feature set.

**Table 2:** Selected skin lesion image datasets and their respective ratio of training to testing.

Dataset	Total Images	Training/Validation Set	Testing Set
PH^2^	200	160	40
ISIC MSK-2	287	201	86
ISIC UDA-1	387	271	116

**Table 3:** Selected classifiers and their functional parameters.

Classifier (Selected)	Base Parameters
Linear SVM	Kernel function: linear. Multi-class method: one-vs-one.
Efficient L-SVM	Kernel function: linear. Multi-class method: one-vs-one.
Cubic SVM	Kernel function: cubic. Multi-class method: one-vs-one.
Fine KNN	Number of neighbors: 1. Distance metric: Euclidean. Weight: equal.
Medium KNN	Number of neighbors: 10. Distance metric: Euclidean. Weight: equal.
Weighted KNN	Number of neighbors: 10. Distance metric: Euclidean. Weight: squared inverse.
Ensemble-BT	Ensemble method: AdaBoost. Learner type: decision tree. Max. split: 20. Number of learners: 30.
Ensemble S-KNN	Ensemble method: subspace. Learner type: nearest neighbor. Number of learners: 30.
Ensemble RUSB	Ensemble method: RUBoost. Learner type: decision tree. Number of learners: 30. Max. split: 20.

**Table 4:** The chosen set of feature vectors and their respective dimensions, along with the percentage of reduction achieved.

Vector Fusion	Input Dimension	Output Dimension	Reduction Percentage (%)
PH^2^			
FV2–FV3	140 × 2562	140 × 948	63
FV3–FV4	140 × 2946	140 × 884	70
FV2–FV4	140 × 3456	140 × 380	89
FV2–FV3–FV4	140 × 4482	140 × 583	87
FV1–FV2–FV3–FV4	140 × 4484	140 × 628	88
ISIC–MSK			
FV2–FV3	201 × 2562	201 × 589	77
FV3–FV4	201 × 2946	201 × 295	90
FV2–FV4	201 × 3456	201 × 242	93
FV2–FV3–FV4	201 × 4482	201 × 403	91
FV1–FV2–FV3–FV4	201 × 4484	201 × 179	96 *
ISIC–UDA			
FV2–FV3	271 × 2562	271 × 436	83
FV3–FV4	271 × 2946	271 × 383	87
FV2–FV4	271 × 3456	271 × 346	90
FV2–FV3–FV4	271 × 4482	271 × 538	88
FV1–FV2–FV3–FV4	271 × 4484	271 × 448	90

*: maximum accuracy achieved.

**Table 5:** Performance comparison of various classifiers over selected datasets.

Classifier	Dataset			Performance Measures					
	**I**	**II**	**III**	**Accuracy (%)**	**Sensitivity**	**Specificity**	**FNR**	**FPR**	**F1 Score**
Linear SVM	✓			88.13	0.833	0.941	0.167	0.058	0.888
		✓		85.11	0.801	0.916	0.198	0.083	0.861
			✓	83.71	0.795	0.885	0.203	0.115	0.845
Q-SVM	✓			87.21	0.843	0.907	0.156	0.092	0.877
		✓		97.12	0.952	0.992	0.048	0.009	0.971
			✓	96.54	0.951	0.979	0.048	0.021	0.965
Cubic SVM	✓			88.52	0.923	0.853	0.076	0.146	0.879
		✓		88.27	0.905	0.866	0.094	0.133	0.882
			✓	87.14	0.893	0.849	0.106	0.154	0.866
Fine KNN	✓			*98.89 * *	0.98	0.989	0.019	0.012	0.985
		✓		*99.01* *	0.985	0.994	0.014	0.005	0.994
			✓	97.71	0.974	0.984	0.029	0.015	0.977
Medium KNN	✓			94.34	0.931	0.949	0.068	0.051	0.941
		✓		93.18	0.921	0.938	0.078	0.061	0.930
			✓	90.55	0.885	0.926	0.114	0.073	0.907
Weighted KNN	✓			87.15	0.862	0.876	0.137	0.124	0.871
		✓		81.39	0.803	0.816	0.196	0.183	0.811
			✓	79.64	0.792	0.798	0.207	0.202	0.796
Ensemble- BT	✓			73.89	0.728	0.742	0.271	0.257	0.738
		✓		75.24	0.745	0.755	0.254	0.244	0.752
			✓	77.38	0.777	0.772	0.222	0.227	0.773
Ensemble S-KNN	✓			97.58	0.978	0.979	0.029	0.025	0.975
		✓		95.46	0.959	0.954	0.04	0.049	0.095
			✓	*99.09* *	0.986	0.994	0.013	0.006	0.993
Ensemble RUSB	✓			95.76	0.952	0.961	0.047	0.038	0.957
		✓		94.89	0.945	0.951	0.054	0.048	0.948
			✓	93.57	0.932	0.939	0.069	0.063	0.935

*: maximum accuracy achieved.

**Table 6:** Overall accuracy comparison of simple fusion approach with the proposed framework.

Vector Fusion	OA (%)					
	**Feature Fusion Approach**			**Proposed Feature Selection Approach**		
	**Q-SVM**	**Fine KNN**	**ES-KNN**	**Q-SVM**	**Fine KNN**	**ES-KNN**
PH^2^						
FV2–FV3	84.31	74.27	74.13	86.23	88.21	81.37
FV3–FV4	79.23	78.34	81.20	88.76	86.33	88.90
FV2–FV4	81.23	81.29	79.45	84.01	88.69	87.54
FV2–FV3–FV4	83.71	79.36	84.56	86.66	92.27	87.43
FV1–FV2–FV3–FV4	83.21	85.22	87.69	87.21	*98.89* *	97.58
ISIC–MSK						
FV2–FV3	74.63	82.27	78.27	79.21	81.89	87.23
FV3–FV4	76.38	83.27	81.17	83.34	81.44	89.28
FV2–FV4	76.31	79.28	76.84	81.23	87.38	84.38
FV2–FV3–FV4	79.48	80.14	79.28	84.27	88.27	90.29
FV1–FV2–FV3–FV4	81.29	81.23	83.73	97.12	*99.01* *	95.46
ISIC–UDA						
FV2–FV3	77.94	79.54	81.24	85.23	85.27	87.07
FV3–FV4	76.28	81.88	82.13	84.36	88.34	89.69
FV2–FV4	81.56	83.29	84.63	88.28	91.26	84.26
FV2–FV3–FV4	83.16	81.83	87.76	89.31	94.18	94.61
FV1–FV2–FV3–FV4	89.74	86.47	87.90	96.54	97.71	*99.09* *

*: maximum accuracy achieved.

**Table 7:** Performance comparison of existing algorithms.

Author	Accuracy
Attique et al. [49]	98.70%
El-Khatib et al. [50]	96.00%
Alizadeh et al. [51]	97.50%
Akram et al. [3]	98.80%
Chatterjee et al. [52]	97.86%
Hameed et al. [33]	97.50%
Khan et al. [53]	98.46%
**Proposed **	**98.89%**

## Data Availability

PH2 dataset: https://www.kaggle.com/datasets/synked/ph2-modified (accessed on 4 August 2023). ISIC-MSK and ISIC-UDA: https://challenge.isic-archive.com/data/ (accessed on 4 August 2023).
